# Surgical mitral valve replacement using direct implantation of Sapien 3 valve in a patients with severe mitral annular calcification without adjunctive techniques, a case report

**DOI:** 10.1186/s13019-020-1083-8

**Published:** 2020-02-24

**Authors:** Turki B. Albacker, Bakir Bakir, Ahmed Eldemerdash, Fayez Elshaer, Hanan Albacker, Murtadha Alawami, Tariq Kashour

**Affiliations:** Cardiac Sciences Department, King Fahad Cardiac Center, College of Medicine, King Saud University, Riyadh, Saudi Arabia

**Keywords:** Mitral valve replacement, Sapien 3 valve, MAC, Direct implantation

## Abstract

**Background:**

Mitral annular calcification (MAC) occurs represents a challenge to surgeons during mitral valve (MV) surgery with increased perioperative risk.

**Case presentation:**

We describe a challenging case of an elderly female patient with multiple comorbidities who presented with symptoms and signs of heart failure with a previous history of mechanical aortic valve replacement 15 years prior to presentation. Echocardiogram showed severe mitral stenosis and regurgitation with severe calcification of the mitral annulus. Given her high-risk profile and unavailability of suitable percutaneous therapeutic options we decided to replace her mitral valve with Sapien 3 valve under direct exposure. The case describes the technical details for the valve implantation and demonstrates the viability of this option in high risk surgical patients without the need for adjunct techniques like predilatation, additional supporting sutures or patches with a review of the literature on open surgical implantation of Sapien 3 valve.

**Conclusion:**

Direct open surgical implantation of Sapien 3 valve can be implanted safely in patients with severe MAC, without predilatation and without the use of other adjunctive techniques like fixation sutures or patches.

## Background

Mitral annular calcification (MAC) occurs in 10% of the population with increasing prevalence with advancing age (up to 40% above 80 years) [1, 2] and it is independently associated with all-cause mortality [3]. It represents a challenge to surgeons during mitral valve (MV) surgery with up to 6 folds increase in perioperative risk [4]. Unfortunately, percutaneous interventions in this high-risk group of patients still carry a higher risk than open surgery with 30-day mortality of 25% and 1-year mortality of 53.7% [5, 6].

We report in this paper a case of open surgery for mitral valve replacement (MVR) in a high-risk patient with severe MAC using the direct implantation of Sapien 3 valve that is designed for percutaneous use without the use of any adjunctive techniques and we reviewed the literature in this subject. This is the first case implanted directly without the use of fixation sutures or any other adjunctive techniques.

## Case presentation

This is a case of a 75-year-old lady who is known to have diabetes mellitus, hypertension, hypothyroidism, adrenal suppression, previous history of stroke and a history of aortic valve replacement with mechanical prosthesis 15 years prior to presentation. She was admitted urgently to the cardiac care unit with shortness of breath on exertion class III and lower limb edema. Chest X-Ray was compatible with pulmonary edema. Echocardiogram showed a well-functioning aortic prosthesis but severe mitral valve stenosis (MS) and mitral regurgitation (MR) with severe Mitral annular calcification (MAC) (video 1, 2).


**Additional file 2 Video 2**: Preoperative 3D Echocardiogram.


The patient was stabilized medically then underwent Cardiac CT to characterize the MAC that appeared as a dense horseshoe calcification occupying most of the circumference of the mitral annulus (Video 3, 4). The case was discussed in the multidisciplinary rounds and she was deemed very high risk for surgical intervention with a calculated STS risk score of (19.5%). Given the patient age and frailty, it was not reasonable to decalcify the mitral annulus and reconstruct the annulus that may lead to high risk of Atrio-ventricular (AV) groove dissociation. So the decision was taken to implant the Sapien 3 valve in the mitral position under direct vision after redo sternotomy. To predict the risk of post procedure LVOT obstruction, the neo LVOT was assessed preoperatively during systole by measuring the distance from the inter- ventricular septum to the frame of the simulated transcatheter valve and then calculating the neo LVOT area which was 211 mm^2^ in this case. However, there is no set threshold for LVOT obstruction when this procedure is done using transatrial approach and the anterior mitral leaflet is excised since the calculated neo LVOT is underestimated due to the fact that there is flow through the cells of the valve stent frame.

Intraoperatively, and after exposing the mitral valve, the anterior leaflet was excised and was thickened and fibrosed (Video 5). The posterior calcium bar was so prominent that made the MV orifice very small not even accommodating size 25 regular MV prosthesis sizer. We decided preoperatively to use the largest Sapien 3 valve (size 29) based on the mitral orifice size from CT scan with additional 20% oversizing. We loaded the valve on the balloon in a similar way to the transapical aortic approach and we advanced the balloon inside the ventricle until the end of the valve stents was just across the mitral annulus (Video 6). We elected not to perform a balloon predilatation to avoid fracture of the calcium body or to induce atrioventricular groove rupture. Subsequently one operator was responsible for inflating the balloon and another one was responsible for stabilizing the position of the valve across the annulus with fine tuning the direction of the valve during inflation of the balloon. The balloon was inflated until an atmospheric pressure of 4 and kept inflated for 15 s then deflated (Video 7). The valve was nicely deployed and stable in position with normal movement of the leaflets. We did not use supportive sutures to fix the valve nor utilized any patches or skirts around the valve. The patient was weaned smoothly from bypass and intraoperative transesophageal echo was performed and showed that the valve is well seated in position with no evidence of any paravalvular leak (Video 8, 9). The mean gradient across the valve was 4 mmHg.


**Additional file 5 Video 5**: Excision of the Anterior Mitral Leaflet.



**Additional file 6 Video 6**: Valve Positioning Across the Mitral Annulus.



**Additional file 7 Video 7**: Valve Deployment over a Balloon.



**Additional file 9 Video 9**: Postoperative 3D Echocardiogram.


## Discussion

Surgery for MVR carries very high risk in patients with MAC and percutaneous MVR techniques did not result in lower risk in this group of patients given its limitations in implantation techniques and the risks of paravalvular leaks and Left ventricular outflow tract (LVOT) obstruction. Hence there is a great need for further development in this field.

The first world-wide case of open antegrade placement of transcatheter valve for MVR was reported by Thierry Carrel et al. in June, 2012. They used SAPIEN XT size 26 valve in an 81 years old woman with severe MR & moderate MS. They decided to use this innovative method to avoid debridement of severe MAC and also due to small annulus, only 19 mm sizer were able to pass after resection of the anterior and part of the posterior leaflet [7]. Since that time only 36 cases were reported in the literature using open antegrade placement of transcatheter valve for MVR (Table 1). All these cases were performed using additional adjunctive techniques for implantation including balloon pre-dilatation, suture fixation, Teflon skirts around the valve and patches around the valve and sutured to the left atrial wall.

**Table 1:** Literature Summary of Open Antegrade Placement of Transcatheter Valve for Mitral Valve Replacement

	Author	year	No. of cases	Age/ Sex	Type of valve	Valve size	Redo	Access	MAC	indication	Note
1	Carrel et al. [7]	2012	1	81/F	Sapien XT	26	yes	Sternotomy	yes	Severe MR/ Moderate MS	1st case
2	Astarci et al. [8]	2013	1	62/F	Sapien XT	26	No	Sternotomy	yes	Severe MS/ Moderate MR	AVR + MVR+ CABG
3	Ferrari et al. [9]	2014	1	60/M	Sapien XT	29	No	Rt Thoracotomy	Yes	Severe MS	Hx of chest radiation Hx of TAVI
4	Lee et. Al [10]	2016	1	83/F	Sapien XT	29	No	Sternotomy	Yes	Severe MR	
5	Murashita et. Al [11]	2016	1	71/F	Sapien XT	29	No	Sternotomy	Yes	Severe MS	
6	Baumgarten et al. [12]	2016	3	1. 89/F 2. 83/F 3. 85/F	1. Sapien XT 2. Sapien XT 3. Sapien 3	1. 26 2. 26 3. 29	1. Yes 2. No 3. No	Mini Thoracotomy	1. Yes 2. Yes 3. Yes	1. Severe MS 2. Severe MS 3. Severe MS	Patient 3 had mild post op PVL
7	Langhammer et al. [13]	2017	4	1. 80/F 2. 60/M 3. 79/F 4. 74/F	1. Sapien XT 2. Sapien XT 3. Sapien XT 4. Sapien 3	1. 26 2. 29 3. 29 4. 29	1. No 2. Yes 3. No 4. No	Sternotomy	1. Yes 2. Yes 3. Yes 4. Yes	1. Severe MR/ Moderate MS 2. Severe MS 3. Severe MR/ Moderate MS 4. Severe MR/ Moderate MS	1. Concomitant Maze, mild PVL 2. – 3. Concomitant CABG/myomectomy 4. Post op mild transvalvular leak
8	Alfonsi et. al [14].	2017	1	76/F	Sapien XT	26	No	Sternotomy	Yes	Severe MS	5. Post op mild PVL
9	Koehle et al. [15]	2017	1	66/ F	Sapien XT	26	yes	Sternotomy	No	Severe MS	Inserted inside a mechanical prosthesis ring
10	Polomsky et al. [16]	2017	2	1. 81/ M 2. 69/ F	Sapien 3	1. 26 2. 26	No	Sternotomy	Yes	1. Mixed 2. Severe MS	
11	Gallo et al. [17]	2018	1	73/F	Sapien 3	29	No	Sternotomy	Yes	Severe MS	
12	Russell et al. [18]	2018	8	1. 65/M 2. 78/F 3. 74/M 4. 87/F 5. 80/M 6. 77/M 7. 75/F 8. 69/F	Sapien 3 (all)	1. 29 2 .29 3. 29 4. 29 5. 29 6. 29 7. 26 8. 26	1. Yes 2. Yes 3. Yes 4. No 5. Yes 6. Yes 7. No 8. No	Sternotomy/ Thoracotomy	Yes (all)	NA	PVL immediately post-implantation was none or trace in 6 patients and mild in 1. There were no cases of moderate or severe PVL. One patient with mild PVL post-TMVR developed hemolysis 6 months post-TMVR that was successfully treated with percutaneous closure using a vascular plug. There were no procedural major complications, including clinically significant LVOT obstruction, annular rupture, valve embolization, or migration. The mean length of stay has been 7.9 days following surgery. There were no in-hospital or 30-day mortalities. No patient had a stroke. One patient (#5) died at home 7 months post-operatively; all other patients are alive.
13	Tabachnick et al. [19]	2018	10	1. 87/F 2. 84/F 3. 86/F 4. 87/F 5. 77/F 6. 71/F 7. 76/F 8. 70/F 9. 83/F 10. 80/F ? details 80/F	1. Sapien XT 2. Sapien 3 3. Sapien 3 4. Sapien 3 5. Sapien 3 6. Sapien XT 7. Sapien XT 8. Sapien XT 9. Sapien 3 10. Sapien XT	1. 26 2. 26 3. 29 4. 29 5. 26 6. 26 7. 26 8. 26 9. 29 10. 29	NA	Mini Thoracotomy/Robotic	Yes	1. Stenosis 2. Mixed 3. Mixed 4. Mixed 5. Stenosis 6. Stenosis 7. Mixed 8. Stenosis 9. Mixed 10. Mixed	
14	Ahmad et al. [20]	2019	1	68/ F	Sapien XT	26	No	Sternotomy	Yes	Severe MS	

The direct surgical implantation of the percutaneous valves has some potential benefits including better orientation of the valve with accurate control of the device depth that may results in lower risk of paravalvular leak. It also allows for excision of the native leaflets that eliminates the risk of LVOT obstruction and decreases the risk of embolization.

In our case, we elected not use balloon pre-dilatation of the annulus in order to avoid the risk of calcium disruption or annular tear. We also wanted to avoid oversizing of the MV orifice before the valve deployment that may lead to paravalvular leak. We also decided not to place any additional fixation sutures for the device neither to use any patch around the device in order to shorten the cross-clamp time as much as possible especially in our elderly lady. We used 20% valve oversizing as the only technique to fix the valve in place using the device radial force. This is the first report of direct catheter based mitral valves implantation without adjunctive techniques.

The SITRAL study (Surgical Implantation of TRAnscatheter vaLve in Native Mitral Annular Calcification Study) [21] was designed to establish the safety and feasibility of the SAPIEN 3 valve for severe MS/MR associated with severe calcification in high risk or inoperable patients. It was started on September, 2016 and is estimated to be completed on December, 2019.

## Conclusion

Direct open surgical implantation of Sapien 3 valve can be implanted safely in patients with severe MAC, who are at high risk of complications from decalcification of the mitral annulus, without predilatation and without the use of other adjunctive techniques like fixation sutures or patches. Longer follow up for these valves are needed to show the long-term outcomes of these techniques.

## Supplementary information

**Additional file 1 Video 1**: Preoperative 2D Echocardiogram.

**Additional file 3 Video 3**: Sagittal Reconstruction of Cardiac CT.

**Additional file 4 Video 4**: 3D Reconstruction of Cardiac CT.

**Additional file 8 Video 8**: Postoperative 2D Echocardiogram.

## Data Availability

• Data sharing is not applicable to this article as no datasets were generated or analysed during the current study.

## Abbreviations

AV: Atrio-Ventricular
LVOT: Left Ventricular Outflow Tract
MAC: Mitral Annular Calcification
MR: Mitral Regurgitation
MS: Mitral Stenosis
MV: Mitral Valve
MVR: Mitral Valve Replacement
